# Successful Treatment of a Patient With Brain Metastasis From Ovarian Cancer With BRCA Wild Type Using Niraparib: A Case Report and Review of the Literature

**DOI:** 10.3389/fonc.2022.873198

**Published:** 2022-04-29

**Authors:** Zhenhua Zhang, Muying Xu, Abbas Sakandar, Xiuju Du, Huailin He, Wenfeng He, Dan Li, Qinglian Wen

**Affiliations:** Department of Oncology, Affiliated Hospital of Southwest Medical University, Luzhou, China

**Keywords:** ovarian carcinoma, niraparib, brain metastases, monotherapy, survival, PARP inhibitors, case report

## Abstract

**Background:**

Brain metastases from ovarian cancer are extremely rare and have a very poor prognosis. A multimodal approach (surgery combined with radiotherapy and chemotherapy) yields the best results in reducing neurological symptoms and prolonging survival. Unfortunately, not every patient receives a complete multimodal treatment due to their individual factors. Poly(ADP-ribose) polymerase (PARP) inhibitors have emerged as a maintenance treatment option for recurrent ovarian cancer. Using PARPi may prolong the overall survival in patients with brain metastases and recurrent ovarian cancer.

**Case Presentation:**

We report a case of a female patient with advanced ovarian cancer without any germline or somatic BRCA mutation. After 21 months, after reduction surgery and adjuvant chemotherapy, she was diagnosed with brain metastasis. Due to her physical fitness and economic situation, she did not receive any radiotherapy or chemotherapy but only received surgical debulking of the brain metastasis and niraparib maintenance treatment. Up to now, she has achieved a good treatment response, and the PFS is 29 months.

**Conclusion:**

Based on the response of our patient, PARP inhibitors as a single agent can probably be considered in patients with brain metastasis from ovarian cancer without BRCA mutation who cannot tolerate radiotherapy and chemotherapy.

## Introduction

Ovarian cancer is a leading cause of gynecological cancer death (1). It is highly metastatic, with approximately 58% of patients presenting with distant metastases at the time of diagnosis, and the 5-year survival rate for such patients is only 30% (2). Furthermore, 60–80% of patients with advanced ovarian cancer experience tumor recurrence after treatment (3). Ovarian cancer metastasis often involves the peritoneum and pleura, and liver, lung, and distant lymph node metastasis are also common. The incidence of brain metastasis from ovarian cancer ranges from about 1 to 3% (4). The optimal treatment for brain metastases from ovarian cancer is currently ill-defined because of the rarity and small number of these patients. Furthermore, a combined approach that includes surgical resection, radiation, and chemotherapy has been recommended (5). However, although these therapies are associated with the greatest improvement in survival, not every patient receives a complete multimodal treatment due to their individual factors. PARPi has emerged as a maintenance treatment option that prolongs the time between hemotherapy treatments. PARP inhibitors are approved for maintenance therapy for patients with BRCA-mutated ovarian cancer and platinum-sensitive recurrent ovarian cancer (6). In the NOVA trial, niraparib treatment resulted in maintenance therapy for platinum-sensitive recurrent ovarian cancer with a longer PFS than placebo, even in BRCA wild-type (median PFS, 9.3 vs 3.9 months) (7). Here, we report a case of brain metastasis from ovarian cancer with BRCA wild-type in both germline and somatic treated with only surgery and niraparib for maintenance therapy and got an excellent result.

## Case Presentation

**Figure 1:**
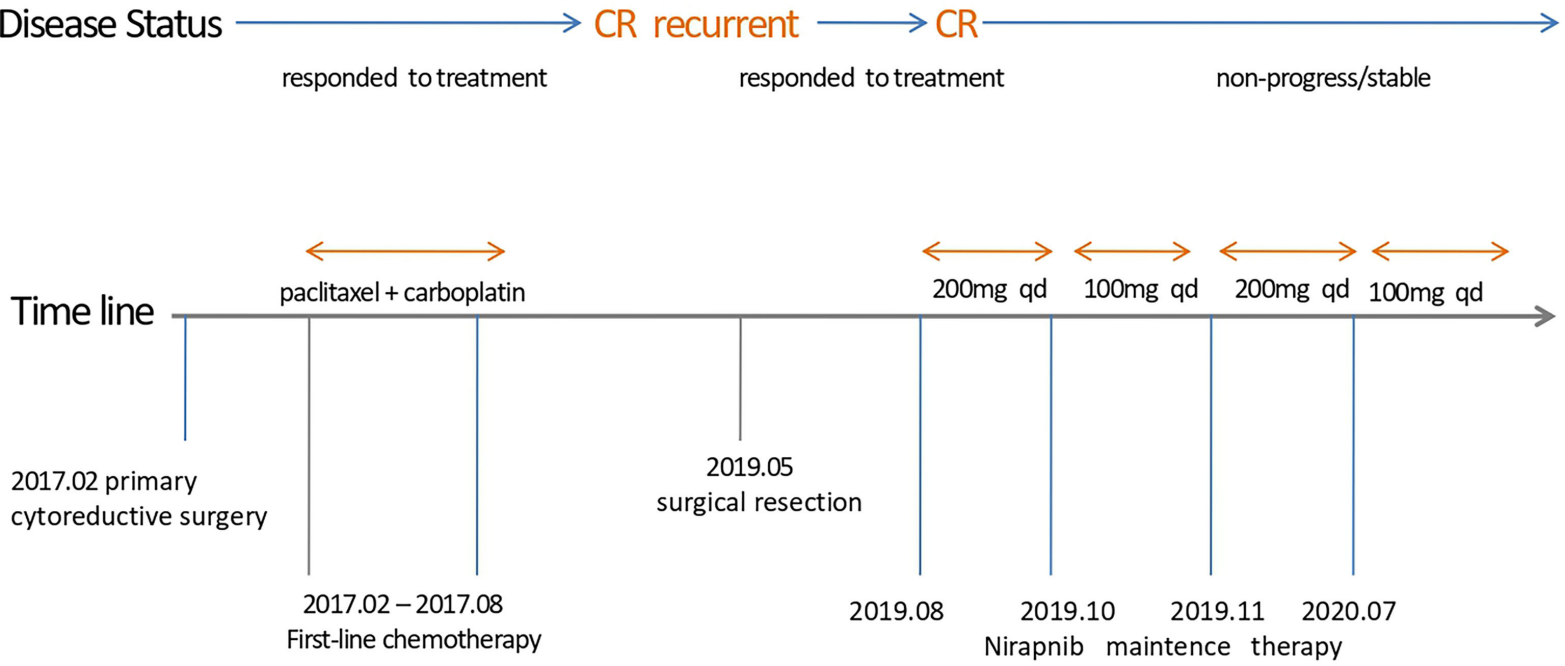
Timeline of different treatments and disease status.

A 48-year-old woman with abdominal pain for one week was admitted to the hospital in Feb 2017 (Sichuan, China). We show the treatment timeline in **Figure 1**. She had epilepsy for more than 20 years and hypertension for several years in her history. In Feb 2017, she underwent primary cytoreductive surgery (R0). Postoperative pathological examination revealed bilateral high-grade serous adenocarcinoma, invading bilateral fallopian tubes, surgical margins of the pelvic wall, uterine serosal surface, the anterior rectal wall mass, peritoneal nodules, appendix, and greater omentum. Three of the 17 left pelvic lymph nodes were found to have metastatic cancer, as were 2 of the 16 right pelvic lymph nodes. Immunohistochemistry (IHC) revealed CK7(+++), CA125(+++), ER(++), PR(+), P53(+++), WT-1(+++), P16(+++), Pax-8(−), and Ki67 (90%+). The postoperative diagnosis was ovarian high-grade serous adenocarcinoma stage IIIC. Her germline and somatic BRCA gene status were BRCA wild type (BRCAwt). After surgery, the patient received adjuvant six-cycle chemotherapy (paclitaxel, 175 mg/m², d1 + carboplatin, AUC = 5, d1, q21d) from 2017.03 to 2017.08. After the treatment, follow-up CT and serum cancer antigen 125 (CA125) revealed no evidence of disease for 21 months.

**Figure 2:**
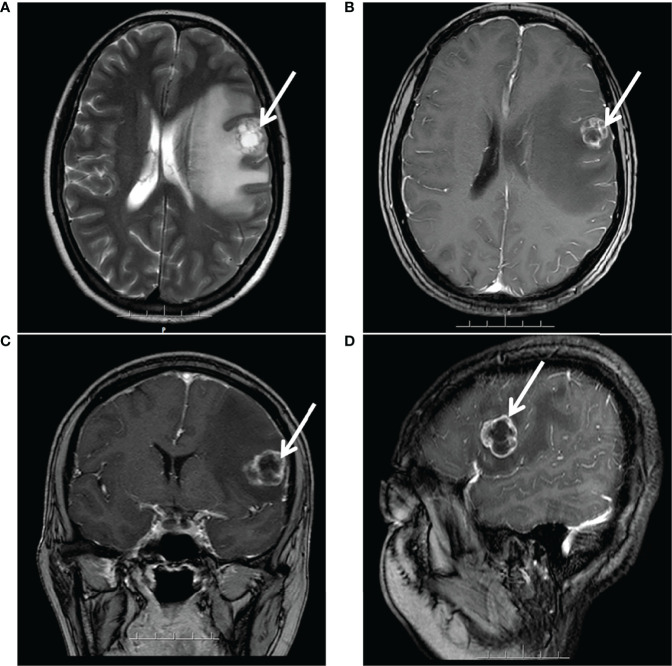
MRI after 21 months of platinum-based chemotherapy: a large prominent tumor in the left frontal lobe with inhomogeneous signal surrounding the tumor mass representing edema. **(A)** Axial T2-weighted. **(B)** Axial gadolinium-enhanced T1-weighted FFE. **(C)** Coronal gadolinium-enhanced T1-weighted FFE. **(D)** Sagittal gadolinium-enhanced T1-FFE.

However, after 21 months, she complained of progressive dizziness and headaches. Due to her medical conditions while in her hometown, she did not undergo any cerebral imaging before dizziness and headaches. Moreover, after a few days, she came to our hospital to complete the examination. Her CA125 level had only 15.5 U/ml, and magnetic resonance imaging (MRI) detected a brain metastasis (a prominent tumor in the left frontal lobe with low-density signal surrounding the tumor mass representing edema) (**Figure 2**), whereas chest-abdominal and pelvic CT revealed no abnormal findings. After combining the history, clinical presentation, and MRI findings of the patient, we concluded that the dizziness and headaches of the patient were related to brain metastases and excluded the possibility of seizures. So no epilepsy imaging was performed. Accordingly, she received surgical resection on 24 May 2019. Postoperative pathological examination showed that the intracranial tissues were metastatic serous ovarian carcinoma. The IHC results were as follows: CK7(+), WT-1(+), P16(+), CA125(+), ER(−), PR(−), Vim (−), Ki-67 (+, 40%), GFAP(−), and CK(+). We were hoping to give brain radiation and chemotherapy, but the patient strongly refused because she thought she could not tolerate chemotherapy and needed medication for her epilepsy. Considering that radiotherapy may aggravate her epilepsy, the patient declined chemotherapy, and she was in complete remission of platinum-sensitive relapse, allowing her to receive PARPi maintenance therapy directly. Moreover, among PARPi drugs, niraparib can cross the blood–brain barrier (BBB) and has no drug-to-drug interaction with her antiepileptics.

**Figure 3:**
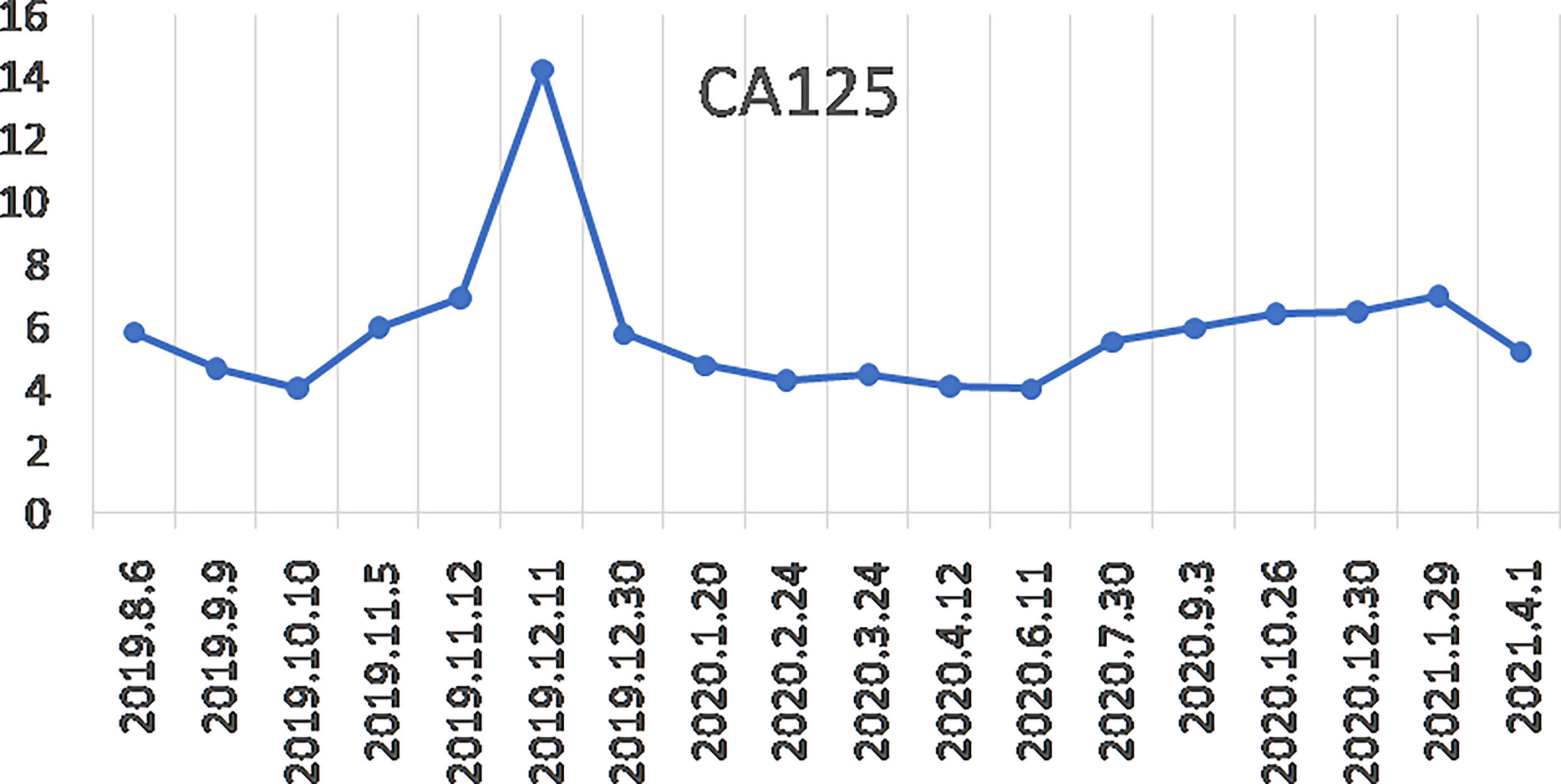
Changes in serum CA125 levels since the start of niraparib treatment.

So, she has been administered niraparib 200 mg once daily since 6 Aug 2019. One week later, she has the side effects of nausea, asthenia, and fatigue. After 11 weeks, the treatment was suspended for four weeks because she developed bone marrow suppression (anemia grade 3, thrombocytopenia grade 2 based on the National Cancer Institute Common Toxicity Criteria for Adverse Events version 4.0) and had a recurrence of epilepsy. After recovering from the bone marrow suppression and epilepsy, the patient resumed niraparib treatment at 100 mg once daily for two weeks. Since Nov 2019, the dosage has been increased to 200 mg once daily. Since July 2020, the dose has been decreased to 100 mg once daily for economic reasons, and the patient is currently on this treatment regimen. During the maintenance treatment with niraparib, her serum CA125 level rose from 5.78 to 14.18 U/ml (6 Aug 2019 to 11 Dec 2019), and then dropped to 5.73 U/ml (30 Dec 2019). So far, it has maintained a low and stable level (3.99–6.94 U/ml) (**Figure 3**). The latest CA125 of the patient was 5.18 U/ml (1 Apr 2021). In the past 29 months after the initiation of niraparib treatment, her KPS score increased to 100, and the head MRI and chest-abdominal-pelvis CT scan revealed no evidence of disease progression. The progression-free survival (PFS) reached 29 months.

## Discussion

Brain metastases are more common in the lung, breast, and malignant melanoma and are considered rare and unfavorable in ovarian cancer, with an incidence of 1–3% (8). The average time from the initial diagnosis of ovarian cancer to the diagnosis of brain metastases is 22 months (9), which is the same as our case. Recently, it has been reported that the incidence of brain metastases from ovarian cancer is on the rise. Firstly, greater awareness among patients and clinicians of this event and improvements in systemic therapies have prolonged survival; secondly, the feature of the first-line chemotherapy drugs paclitaxel and platinum is that they cannot cross the BBB, so they do not possess the potential to reach a high concentration in the cerebrospinal fluid (5); and thirdly, the advancement of imaging techniques such as contrast-enhanced magnetic resonance imaging (MRI) and the newly introduced positron emission tomography/computerized tomography (PET/CT scan) allows for the detection of small metastatic lesions (10, 11).

Currently, due to the rarity of brain metastases from ovarian cancer, there is no consensus regarding the best management of these patients. Through a literature review, several therapeutic approaches can be proposed for brain metastases, namely, surgical resection, radiotherapy (whole-brain radiation therapy, stereotactic radiotherapy, including gamma-knife radiosurgery), and systemic chemotherapy. Borella summarized the treatment results for these patients. Trimodal therapy has obtained the best survival rate, while monotherapy is associated with poor survival. The median OS of surgery combined with radiotherapy ± chemotherapy resulted in better outcomes (23 months and 20 months) compared with surgery (6.5 months) or radiotherapy (5.4 months) or observation (2.4 months) (12). Thus, surgery combined with radiotherapy and chemotherapy has been the best treatment for patients with multiple BM. However, not every patient receives a complete multimodal treatment due to their factors. In addition to these treatments, several inhibitors of PARP have been approved by the Food and Drugs Administration (FDA) for treatment maintenance after adjuvant chemotherapy for ovarian cancer and platinum-sensitive recurrent ovarian cancer. Such inhibitors include olaparib for BRCA mutation carriers, niraparib, and rucaparib for ovarian cancer regardless of BRCA status (7, 13, 14). A portion of the PARPi, like niraparib, can cross the BBB (15, 16). Additionally, Kim et al. revealed higher brain penetration as a unique feature of niraparib compared to other PARPi such as rucaparib, veliparib, talazoparib, and olaparib in both patient and pre-clinical models. However, the response rate in BM patients to these drugs has not been determined (17). Sun showed that brain tumor exposure to niraparib is 3.3 times greater than plasma exposure in tumor xenograft mouse models and shows good sustainability. In comparison, the tumor exposure to olaparib is more petite than in plasma, and sustained brain exposure is not observed. Additionally, niraparib achieves a more potent tumor growth inhibition than olaparib in BRCAwt models and an intracranial tumor model at the maximum tolerated doses (MTD) (18). PARPi could also play a role in treating brain metastases from ovarian cancer. Seven cases have been reported, despite the current lack of data in a large series of patients (**Table 1**). In most of those cases, the PFS was over 12 months. Although these cases remain anecdotal, the results are encouraging, and there is a solid pre-clinical rationale supporting the use of PARPi in these patients, especially in those with BRCA mutated tumors.

**Table 1:** Seven cases of successful treatment for brain metastases from ovarian cancer with PARP inhibitors.

Authors & Year	FIGOStage	Numberof Brain metastases	Extracranial Lesions	Gene Mutation	Treatment strategy	PFS
Bangham et al. (19)	IVB	1	None	BRCA2	Surgery + radiotherapy + chemotherapy + olaparib	12 months
Gray et al. (20)	IIIC	>2	None	BRCA1	Radiotherapy + chemotherapy + niraparib	22 months
Favier et al. (21)	IIIC	Multiple	Peritoneum	BRCA2	Radiotherapy + chemotherapy + olaparib	14 months
Vásquez et al. (22)	IVA	2	None	BRCA1	Chemotherapy + surgery + radiotherapy + olaparib	9 months
Tao et al. (23)	IIIC	Multiple	Peritoneum	BRCA2	Chemotherapy + radiotherapy + niraparib	>15 months
Kashermanet al. (24)	Unknown	Multiple	None	BRCA1	Radiotherapy + chemotherapy + olaparib	11 months
Ikuko Sakamoto et al. (25)	IIIC	Multiple	None	BRCA1	Chemotherapy + radiotherapy + olaparib	>18 months

As far as we are aware, this is the first report on the efficacy of niraparib monotherapy in a Chinese patient with a single brain metastasis from ovarian cancer with BRCAwt. After three months of surgery, the patient did not undergo any radiotherapy or chemotherapy, but niraparib treatment. She has maintained excellent functional status without disease progression for more than two years (up to now) after the onset of brain metastasis, whereas the reported OS of brain metastasis treatment with surgery was only 6.5 months. Furthermore, the dose of niraparib was reduced to 200 mg once daily, as the guideline-recommended 300 mg once daily at that time, which is the same as the later NORA study (26). After the patient received the dose for one year, the dose was reduced again to 100 mg once daily for economic reasons, but still maintained with no disease progression and a low level of CA125. Admittedly, the BRCA1/2 status of the patient is wild type. Stasenko showed that in the brain metastases of ovarian cancer, the BRCA mutation-negative was 70%, whereas the BRCA mutation was 33%, and patients with the BRCR mutation are more likely to have brain metastases than patients without a BRCR mutation. Moreover, patients with BRCR1/2 mutations treated with PARPi have a longer median PFS than patients without the mutation (27). Our patient also has a prolonged PFS even though she has BRCAwt.

Nevertheless, this case report is limited to having not the HRD status for any conditions to test in that year.

## Conclusion

Ovarian cancer brain metastases are rare events with a poor prognosis. Patients need multimodal treatment, namely, surgery, radiotherapy, and chemotherapy. PARPi is recommended for maintenance in platinum-sensitive recurrent ovarian cancer, but there are few reports of its efficacy in brain metastases from ovarian cancer. In our case, the patient underwent surgery without radiotherapy and chemotherapy, but only niraparib as maintenance therapy with a low dose, and then presented a very considerable clinical response even if the patient was without BRCR mutation. The encouraging result should be confirmed in clinical practice for handling similar patients.

## Data Availability Statement

The original contributions generated for the study are included in the article. Further inquiries can be directed to the corresponding author.

## Ethics Statement

Written informed consent was obtained from the individual(s) for the publication of any potentially identifiable images or data included in this article.

## Author Contributions

ZZ and MX took the lead in drafting the manuscript and provided magnetic resonance images. AS made an outstanding contribution to the article revision process. DL, QW, HH, WH, and XD provided supervision and participated in the literature review and drafting of the manuscript. All authors listed have made a substantial, direct, and intellectual contribution to the work and approved it for publication.

## Conflict of Interest

The authors declare that the research was conducted in the absence of any commercial or financial relationships that could be construed as a potential conflict of interest.

## Publisher’s Note

All claims expressed in this article are solely those of the authors and do not necessarily represent those of their affiliated organizations, or those of the publisher, the editors and the reviewers. Any product that may be evaluated in this article, or claim that may be made by its manufacturer, is not guaranteed or endorsed by the publisher.
